# Extra-oral expression of bitter taste receptors in pigs and their correlation with hepatic cytochrome P450 enzymes

**DOI:** 10.1007/s00441-025-04021-w

**Published:** 2025-11-05

**Authors:** Ewelina Pych, Navid Sahebekhtiari, Bo Ekstrand, Martin Krøyer Rasmussen

**Affiliations:** 1 Department of Food Science, Aarhus University, Aarhus, Denmark; 2 Department of Life Science, Chalmers University of Technology, Gothenburg, Sweden

**Keywords:** G protein-coupled receptors, Bitter taste receptors, TAS2R, Porcine, Gene expression, RT-PCR, RNA-seq, Detoxification, Cytochrome P450, Metabolism

## Abstract

Bitter taste receptors (TAS2Rs), known for their role in oral taste perception, are also expressed in extra-oral tissues, suggesting broader physiological functions. This study examined TAS2R expression in porcine tissues and their potential link to detoxification via cytochrome P450 (CYP) enzymes. Using transcriptome profiling complemented by PCR validation, we examined 14 TAS2Rs and detected 8 receptors across multiple tissues, including tongue, liver, jejunum, duodenum, kidney, colon, ileum, and white adipose. Distinct tissue-specific expression patterns were observed, with the highest diversity and abundance in metabolically active organs. Correlation analysis revealed that TAS2R expression levels were not only strongly associated across tissues but also closely aligned with hepatic CYP content, suggesting a functional relationship. These findings provide novel insights into the extra-oral roles of TAS2Rs in pigs, highlighting their potential involvement in xenobiotic sensing, detoxification, and metabolic regulation.

**Supplementary Information:**

The online version contains supplementary material available at 10.1007/s00441-025-04021-w.

## Introduction

The ability to detect and avoid bitter compounds has long been considered an important mechanism for protecting organisms from environmental threats. This defence is made possible by bitter taste receptors, specialized receptors that enable the recognition of potentially harmful substances (Wooding et al. 2021). Although traditionally associated with the detection of harmful substances in food, TAS2Rs are now recognized as key molecular gatekeepers involved in various extra-oral locations, including gastrointestinal, endocrine or respiratory systems (Bloxham et al. 2020). The perception of bitterness, one of five basic taste attributes, is interpreted as a “warning” for the individual to avoid harmful and toxic compounds originating from food and drinks (Luo et al., 2025). Bitter taste receptors, known as TAS2Rs (type 2 receptors), are members of G protein-coupled receptor (GPCR) family responsible for detecting bitterness on the tongue (Lattin et al. 2007; Ekstrand et al. 2017). The number of TAS2Rs varies significantly across species. In humans, 26 isoforms have been identified (Lang et al. 2023), while mice have 35 (Chandrashekar et al. 2006), and chickens only three (Shi and Zhang 2006). This variation reflects differences in dietary needs and evolutionary adaptations related to taste sensitivity. Studies have shown that activation of extra-oral TAS2Rs plays important physiological roles, regulating appetite and inducing obesity in mice (Avau et al. 2015). The expression of TAS2Rs in metabolically active tissues including the liver, skeletal muscle and adipose tissues, may imply a connection between TAS2R and metabolism (Kimura and Kato 2020). While significant research has been conducted on humans and mice, the porcine models remain understudied, providing a unique opportunity to expand scientific understanding of these receptors in a new biological context. The porcine bitter taste system exhibits a higher frequency of allelic variants compared to non-bitter taste genes, indicating a possible role of these genes in pigs' ecological adaptation (Foster et al., 2014). The chemosensory anatomy of pigs and humans appears comparable, with a similar ratio of taste buds to body weight (Roura et al. 2016). The bitter taste systems of pigs and humans are both diverse and shaped by species-specific evolutionary pressures linked to diet and habitat (Rowland et al. 2015). The similarity in nutrient receptors and taste sensitivities between pigs and humans creates the opportunity to use the pig as a model in place of rodents for behavioural studies into chemosensing (Roura et al. 2016). Pigs and minipigs are highly regarded as models for human studies, especially in safety evaluations of pharmaceuticals, biopharmaceuticals, and chemicals. Their physiological and metabolic similarities to humans, including drug metabolism via the P450 system, have made them valuable alternatives to traditional non-rodent species (Swindle et al. 2012). Notably, the porcine cytochrome P450 enzymes—especially those from families CYP1, CYP2, and CYP3, which are key enzymes in drug metabolism—demonstrate a high degree of similarity to their human counterparts. For instance, porcine CYP2A19 shares approximately 87.5% primary sequences homology with human CYP2A6, and CYP3A29 in pigs is closely related to human CYP3A4 (82.8%), both in terms of structure and functional activity​ (Burkina et al. 2017). Clark et al. (2012) proposed that extra-oral TAS2Rs in humans may contribute to the off-target effects of various pharmaceuticals that also interact with CYP family enzymes. For example, erythromycin activates the bitter taste receptor TAS2R10 while simultaneously acting as an inhibitor of the drug-metabolizing enzyme CYP3A4. Similarly, drugs like chloroquine and haloperidol are known to activate multiple TAS2Rs and are also substrates or inhibitors of CYP enzymes, highlighting a potential overlap between taste receptor signaling and metabolic pathways. Da Silva et al. (2014) identified a repertoire of 14 porcine bitter taste receptors in the circumvallate papillae of the pig's tongue, however, the physiological functions of these receptors, particularly in extraoral tissues remain unknown.

The aim of this study was to investigate the expression of bitter taste receptors in different porcine tissues. In this study, we used real-time-PCR (RT-PCR) and digital droplet PCR (ddPCR) to determine the mRNA expression of bitter taste receptors in the tongue and selected extra-oral tissues (liver, jejunum, duodenum, kidney, ileum, colon, white adipose). Additionally, we employed transcriptomics as an untargeted, exploratory approach to compare receptor expression in the tongue and liver. We also investigated possible link between TAS2R and CYP enzymes regulation, as a possible correlation between bitter taste receptors and metabolism of drugs and detoxification.

## Materials and methods

### Animal material

**Fig. 1:**
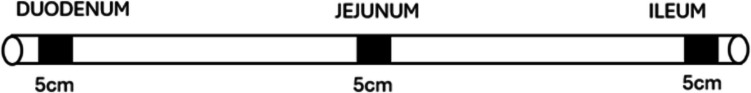
Schematic representation of the small intestine indicating the specific regions (duodenum, jejunum, ileum) from which 5 cm tissue samples were collected

Preliminary data obtained from ddPCR analysis of liver samples in mini pigs (*n* = 5 females, *n* = 5 males), for details see Rasmussen et al. (2019) showed no statistically significant differences between sexes (supplementary Fig. 1). For that reason, only one sex was included in this study. Ten female pigs (crossbreed Landrace x Yorkshire Sire and Duroc boar) weighing 20–30 kg were slaughtered at our research station (AU Viborg, Aarhus University) by trained veterinarians. Upon visual inspection, none of the animals showed any sign of illness or abnormalities. Immediately after killing tissue-samples were collected from tongue, liver, kidney, white adipose (WAT), jejunum, duodenum, colon, and ileum. To investigate the expression and potential metabolic roles of bitter taste receptors, tissues involved in taste perception (tongue), nutrient sensing and absorption (duodenum, jejunum, ileum, colon), and systemic metabolism (liver, kidney, white adipose tissue) were selected. The standardized procedures used for collecting tissue samples from the various organs are outlined in Table 1, while the specific regions of the small intestine (duodenum, jejunum, and ileum) from which intestinal samples were obtained are illustrated in Fig. 1. After dissection, the tissue samples were snap-frozen in liquid nitrogen and stored at –80 °C for subsequent analysis.

**Table 1:** Description of procedure for collecting the samples

Tissue	Procedure of collecting
Tongue	1 cm × 1 cm pieces of epidermis (top layer), not including dermis (connective tissues) or muscle layer
Liver	1 cm × 1 cm pieces from lateral lobes
Kidney	1 cm × 1 cm pieces from cortex
White adipose	1 cm × 1 cm pieces from neck
Jejunum	5 cm of middle section, cut lengthwise and rinsed with water to remove stomach contents
Duodenum	5 cm of initial middle section, cut lengthwise and rinsed with water to remove stomach contents
Colon	5 cm of middle section, cut lengthwise and rinsed with water to remove stomach contents
Ileum	5 cm of final section, cut lengthwise and rinsed with water to remove stomach contents

### RNA isolation and reverse transcription

Total RNA from the collected tissues was extracted using TRIzol LS reagent (Invitrogen, MA, USA) according to the manufacturer’s instructions. Each RNA pellet was resuspended in 30 µL DNase/RNase-free water (Invitrogen, MA, USA). Total RNA concentration was determined by measuring the absorbance at 260 nm, while the purity was estimated from the A260/A280 ratio, using the Nanodrop One spectrophotometer (Thermo Scientific, MA, USA). Reverse transcription was performed using 800 ng of total RNA with the iScript™ cDNA Synthesis Kit (Bio-Rad Laboratories, CA, USA), following the manufacturer’s instructions.

### RT-PCR

TAS2Rs gene sequences were identified from the Sscrofa 10.2 genome version using Ensembl and the NCBI databases. Subsequently, PCR primers and Taqman probes were designed for 14 sequences recognized as porcine homologs of human transcripts using Primer Express 3.0.1 (Thermo Scientific, MA, USA) (Table 2). These primers and probes were custom-made by TAG Copenhagen A/S. RT-PCR was performed in a 10 µL reaction volume containing 2 µL cDNA and iTaq™ Universal Probes Supermix (Bio-Rad Laboratories), following the manufacturer’s instructions. Thermal cycling conditions were: 50 °C for 2 min, 95 °C for 10 min, followed by 40 cycles of 95 °C for 15 s and 60 °C for 60 s. Relative gene expression was normalized to RPLP1 and calculated using the ∆∆Ct method (Schmittgen and Livak 2008).

**Table 2:** The probe and primer sequences used in RT-qPCR. The forward and reverse sequence lists are provided as 5′ − 3′ and used probes are from minor groove binding (MGB) type

Gene	Gene ID	Forward primer	Reverse primer	Probe
TAS2R1	106,508,396	AGAATGGGCAGTGCGTTAGC	TGTGTGTTGATGAAGGGACATCT	CACCCCAGCCCAGCA
TAS2R3	100,621,051	CACTCTGGGTGGCACGAAA	AAAGAAGATGGAGGATTGGACTCA	CCAGCTGCCTTTGAA
TAS2R4	100,620,853	TCCTGGGTCGTGGAGATCAC	CGAACTTCCCACCACTCTTCA	CCTTGGTCCACTGGC
TAS2R7	100,523,246	TGGATGAGGTGGAGAATTGACA	GGGCCAAACACCCTAGCA	TGCAATTCCAAGGATC
TAS2R8	100,523,063	CCCACTTTTGCCCTGGTTTA	CCAGCTGGCTCCAACGAA	TGGAGAATCAGCAGGGTG
TAS2R9	100,522,867	TTATGCCACAGGGTCCAGAGA	ACTGCCTTTATGGCCCTCAA	CTAGCACAGAGGCCCA
TAS2R10	105,499,949	GAGAAATGGGTGGTCCAAGAAA	TGCGAGAAAGAATGCAAGTGA	AAATCCCTGAAGACCTC
TAS2R16	100,513,769	GCTGCCTCTGCGACCTACAC	GGCTTCGCTCAGTGGGTTAA	CTCATGACCATGCTGAATA
TAS2R38	100,624,167	CAAAACTGGGCTTGGAAATTG	AGGGCCTGGCATCACAGA	ACCCTGTCTTGTTTGC
TAS2R39	100,621,890	TGGCCACTCCATCCTACTGAT	CCGCTTCCAAGCTCTTCTCA	CGGGACAACCCTG
TAS2R40	106,508,195	GCTCTCCCACCGAGCCTTA	GAGATCGTCCTGGGCAAACA	AGAGTGACCAGATACCT
TAS2R41	100,524,003	CTCCGAGAGCGACTGGTACTG	TGGACGGATGTGCACAAGTAG	CGTGGCAAATTTTA
TAS2R42	100,156,946	TCACTTCTCCCACTGCCTTTTC	GTATCCCAGGAATGACTTTGTTCA	TCTGGCTGAAGCGGA
TAS2R60	100,524,179	AGCCGTCTACCCCATCGTT	CCTCGCTAGCACAGCTCTCA	TGCTCTTGAGTAACCGC
RPLP1	100,170,119	CGGAGCTCGCCTGCAT	TTATCCTCCGTGACCGTAACCT	ACTCTGCCCTCATTCTGCACGACGA

### Digital droplet PCR

The ddPCR reactions were performed according to the manufacturer’s instruction. Briefly, reaction mixture consisted of 2X ddPCR supermix for probes (no dUTP) (Bio-Rad, USA), 900 nm forward and reverse primers, and 250 nm for each probe and up to 250 nm of cDNA template, to a final volume of 20 µL. Initial concentration of cDNA was 50 ng/µL. The reaction mix was prepared in 96-well plates and was properly mixed and spin down. Nano-droplets were generated using the QX200™ Droplet Generator (Bio-Rad Laboratories). The new plate was then sealed and placed in a C1000 Touch Thermo Cycler, where the PCR protocol was set as follows: 95 °C for 10 min, followed by 40 cycles (94 °C for 30 s and 53,2 °C for 60 s) and 98 °C for 10 min. The optimal annealing temperature was determined by running a temperature gradient (supplementary Fig. 2). Reading of the droplets was done using the QX200™ Droplet Reader (Bio-Rad Laboratories), followed by data analysis using QX Manager™ Software, Standard Edition, Version 1.2 (Bio-Rad Laboratories), in accordance with the manufacturer’s instruction.

### Transcriptome profiling

Transcriptome profiling of liver and tongue tissue using Affymetrix gene arrays was conducted to determine the overall gene expression, following the methodologies outlined by Rasmussen et al. (2023). Briefly, 50 ng of RNA was used for cRNA fragmentation and labelling. The fragmented and labelled cRNA were then loaded onto Porcine-specific gene arrays (Affymetrix PorGene-1_0-st, Thermo Fisher Scientific). Hybridization was carried out for 16 h, after which the arrays were washed using the Fluidic Station 450 s (Thermo Scientific) and scanned with the GeneChip Scanner 3000 7G (Thermo Scientific). The resulting data underwent quality assessment and processing, using the Transcriptome Analysis Console (TAC) software 4.0.2. (Applied Biosystems). Genes were considered differentially expressed if false discovery rate was < 0.05.

### Statistical analysis and data visualization

Data analysis and visualization was performed in GraphPad Prism v10.3.1 software (La Jolla, CA, USA) and R Studio Software. Statistical significance was assessed using a RM Anova, followed by Tukey’s post-hoc test or Friedman test, followed by Dunn’s post hoc test. The p-value of less than 0.05 was considered statistically significant. When appropriate, ROUT method (Q = 1) was applied to identify outliers in GraphPad Prism.

To determine correlation of TAS2Rs within tissue Pearson’s correlation was utilized. All data are presented as means ± standard deviations.

## Results

### Characterization of bitter taste receptors in porcine tissues

**Fig. 2:**
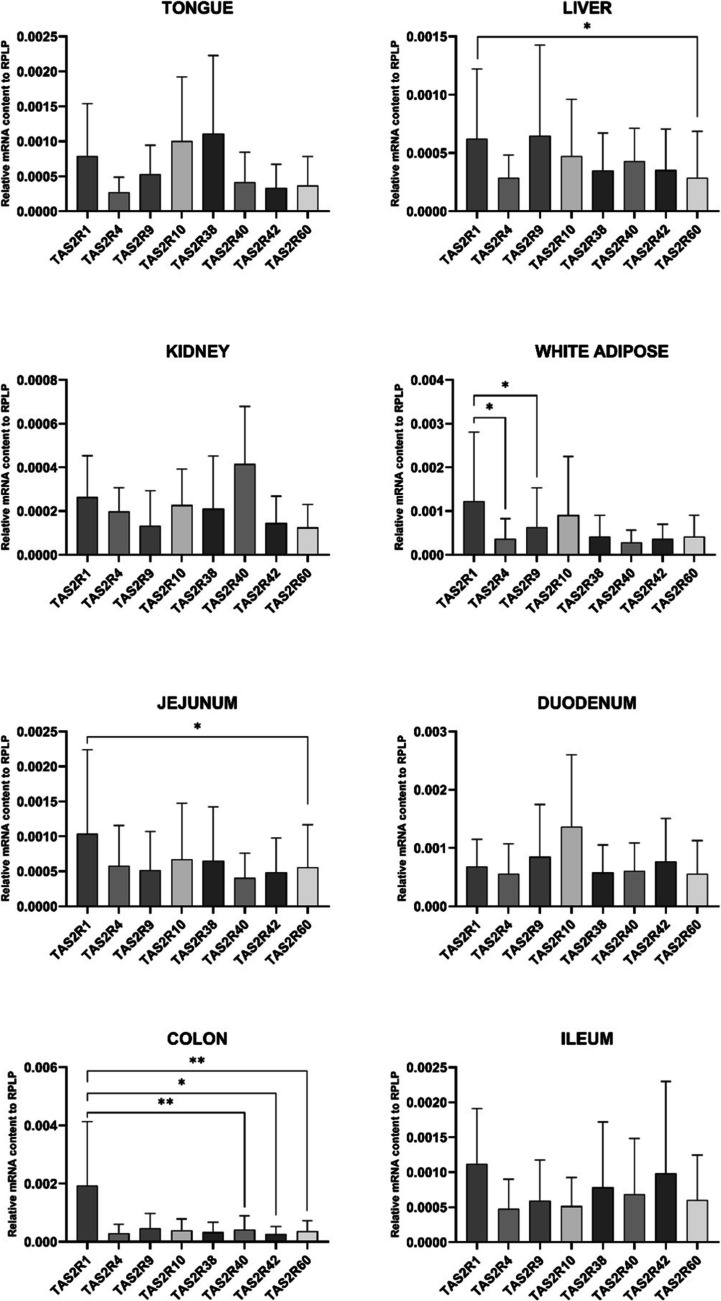
mRNA expression levels of bitter taste receptors measured in porcine tissues (*n* = 5–10). Only mRNA amplified in at least 5 out of 10 donors is shown in the graph. Data are presented as mean ± standard deviation. Statistical differences between tissues were assessed using a RM ANOVA, followed by Tukey’s post hoc test. *p < 0.05, **p < 0.01

We determined the content of bitter taste receptors of each of the eight different tissues, the relative mRNA content, within tissue of TAS2R1, TAS2R3, TAS2R4, TAS2R7, TAS2R8, TAS2R9, TAS2R10, TAS2R16, TAS2R38, TAS2R39, TAS2R40, TAS2R41, TAS2R42, TAS2R60 using RT-PCR. We observed amplification for 8 out of 14 target genes chosen for the study (Fig. 2). For all the expressed genes, Ct values were < 34, suggesting the presence of moderate quantities of the target mRNA. Notably, we observed significant differences in the liver between TAS2R1 and TAS2R60; in the white adipose tissue between TAS2R1 and TAS2R4, and TAS2R1 and TAS2R9; in the jejunum between TAS2R1 and TAS2R60; and the colon between TAS2R1 and TAS2R40, TAS2R1 and TAS2R42; and TAS2R1 and TAS2R60.

**Fig. 3:**
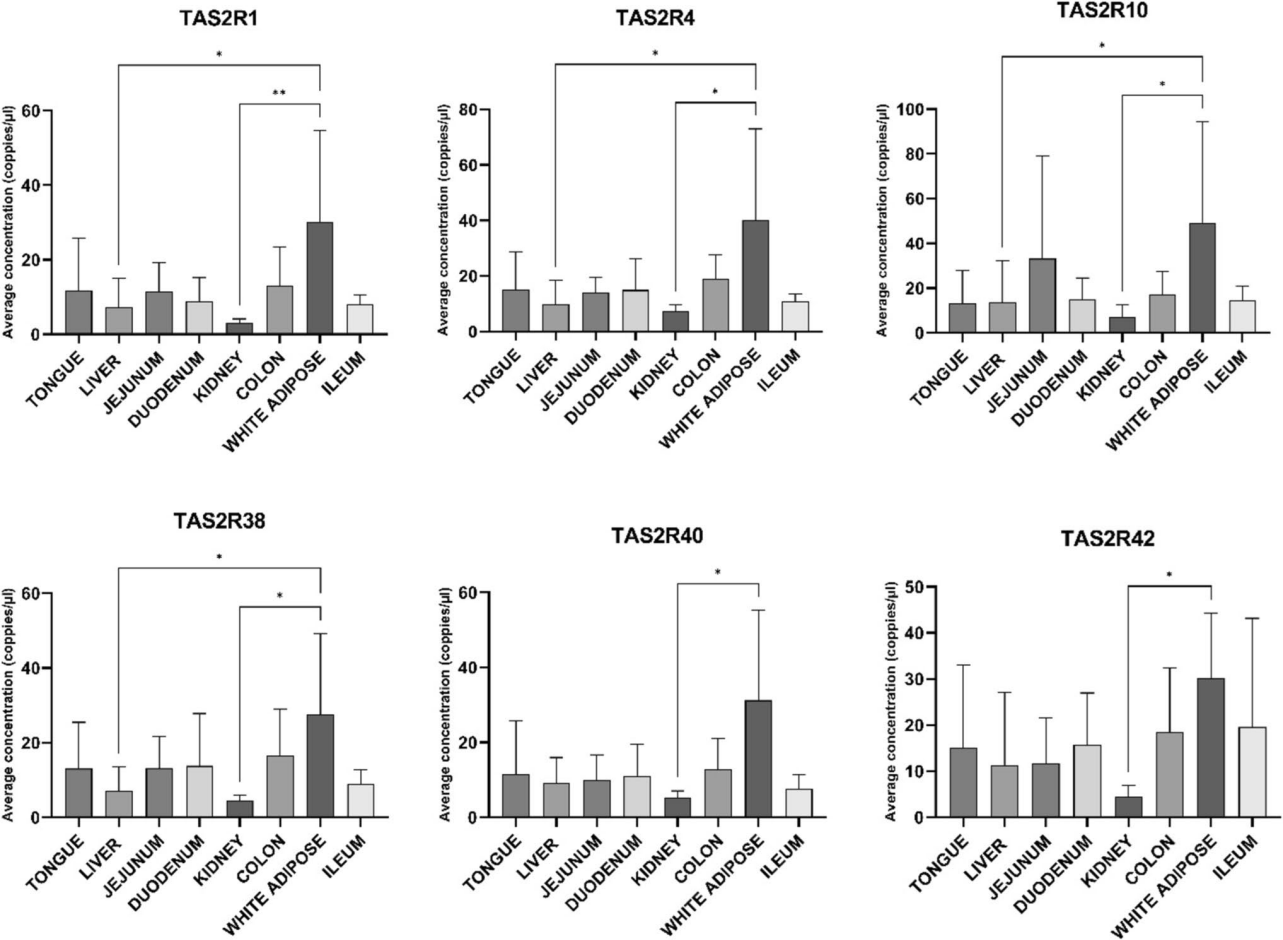
Average mRNA concentration (copies/µL) of bitter taste receptors in 8 different porcine tissues (*n* = 6). Data are presented as mean ± standard deviation. Statistical differences between expression across tissues were assessed using a Friedman test, followed by Dunn’s post hoc test. *p < 0.05, ** p < 0.001

To compare the expression levels of specific bitter taste receptors across tissues, we utilized ddPCR. This method enabled accurate analysis without the need for a reference gene with stable expression, which is challenging to establish across distinct tissues (Kuhlmann et al. 2021). Expression levels of six selected TAS2Rs were analysed across all examined tissues (Fig. 3). Significant differences in tissue expression were observed between WAT and liver, as well as between WAT and kidney for TAS2R1, TAS2R4, TAS2R10, and TAS2R38. Additionally, TAS2R40 and TAS2R42 showed significant differences between WAT and kidney.

### Cross-tissue correlation of TAS2Rs

**Fig. 4:**
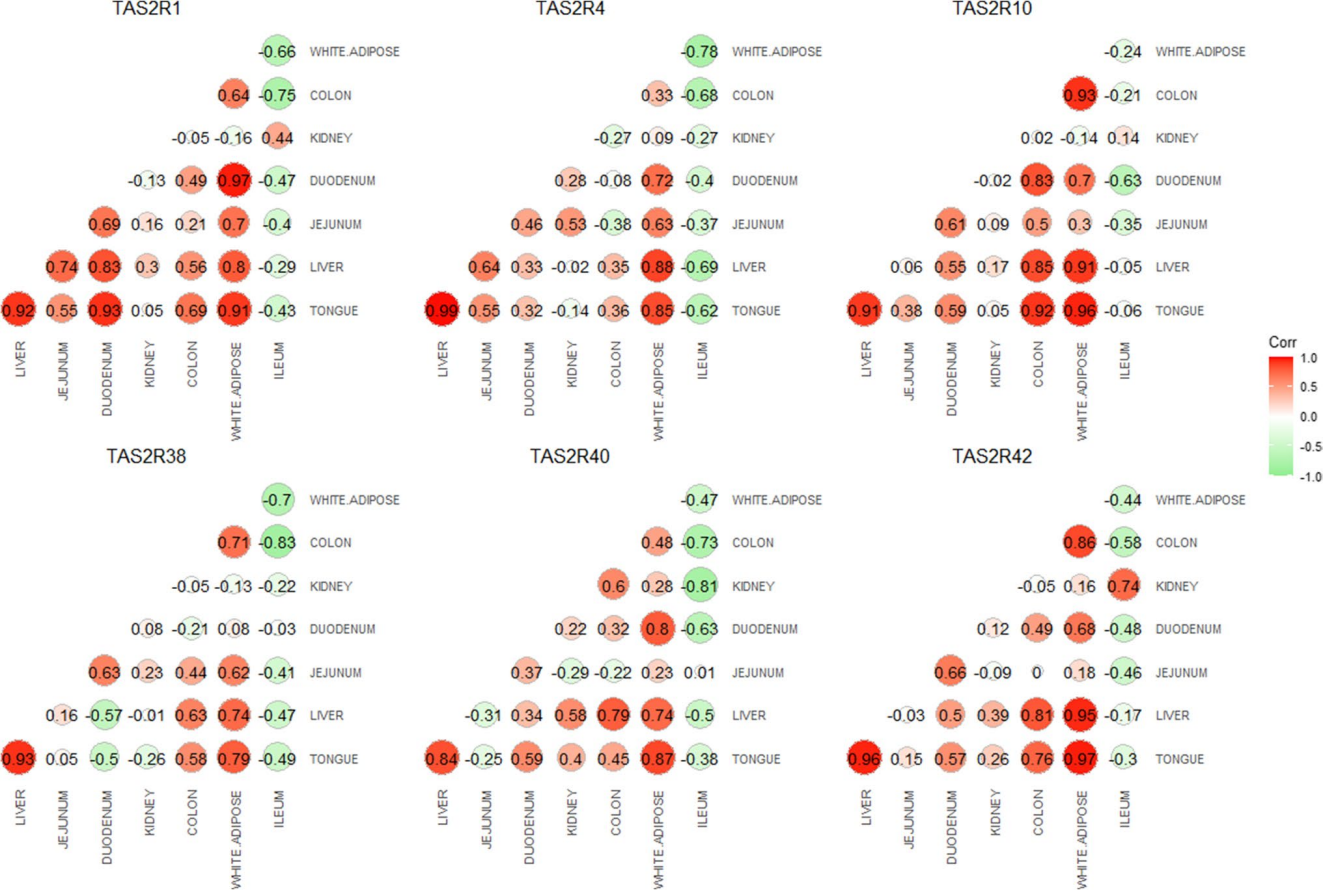
Pearson’s correlation for six TAS2Rs based on ddPCR results (*n* = 6). Values presented in the plots represent correlation coefficients

To investigate whether TAS2R expression levels are correlated between tissues, we used Pearson’s correlation to assess the degree of similarity in their expression patterns. Correlograms illustrate the correlation coefficients between the expression levels of six TAS2R receptors, as measured by ddPCR (Fig. 4).

The correlation matrices demonstrated strong positive correlations for TAS2R1, TAS2R4, TAS2R10, TAS2R38, TAS2R40, and TAS2R42 across several tissues. Specifically, all six receptors showed significant correlations between the tongue and liver, tongue and WAT, as well as liver and WAT. Additionally, TAS2R1 exhibited a strong positive correlation between the tongue and duodenum. In contrast, we observed strong negative correlations between the ileum and colon, and between the ileum and WAT, for all receptors except TAS2R10.

### Transcriptome profiling

**Fig. 5:**
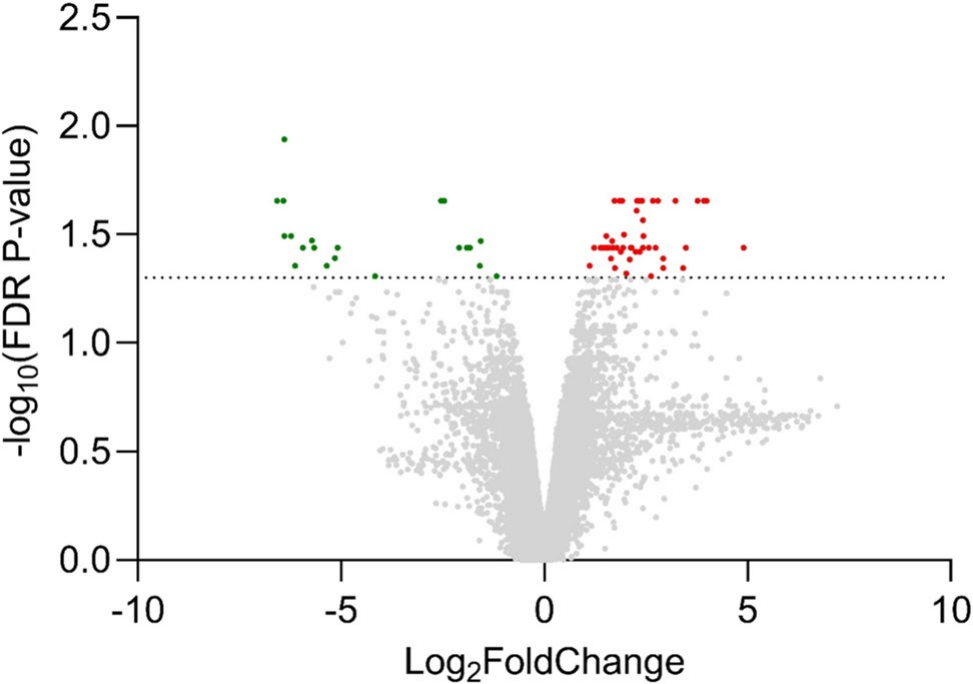
Volcano plot for transcriptomics data representing difference in gene expression between tongue (*n* = 4) and liver (*n* = 4). The volcano plot displays log_2_(fold change) on x-axis and -log_10_(FDR p-value) on y-axis, highlighting significant up-regulated (red) and down-regulated (green) genes

We employed an untargeted transcriptomic approach to further investigate the expression of TAS2Rs and related genes, including those implicated in potential signaling pathways. This analysis included four liver and four tongue samples. In total we identified 71 transcripts with significantly different expression between the liver and tongue (FDR p-value < 0.05). Of these, 49 transcripts were upregulated, and 22 were downregulated (Fig. 5).

**Fig. 6:**
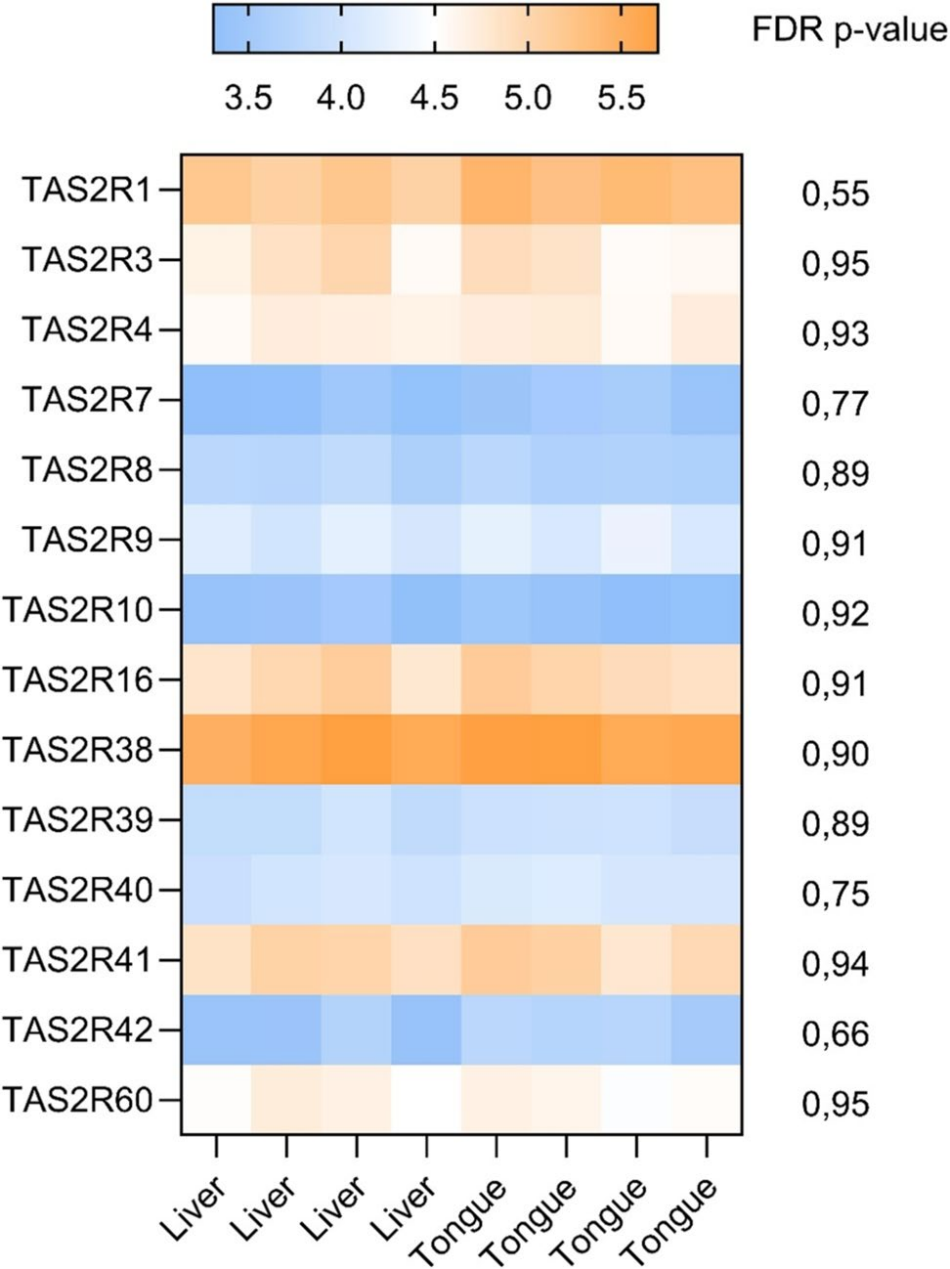
Heat map of sample signal obtained from transcriptomics for TAS2Rs in both liver and tongue tissue from the same donors (*n* = 4 for tongue, *n* = 4 for liver) with indicated FDR *p*-value

The analysis identified the presence of 14 transcripts for bitter taste receptors; however, none of them was differently expressed (Fig. 6). TAS2R1, TAS2R16, TAS2R38 and TAS2R41 were predominantly expressed in both tissues. The lowest signal was observed for TAS2R7 and TAS2R10.

### Transcriptomic co-expression analysis of TAS2Rs and CYP450 isoforms

**Fig. 7:**
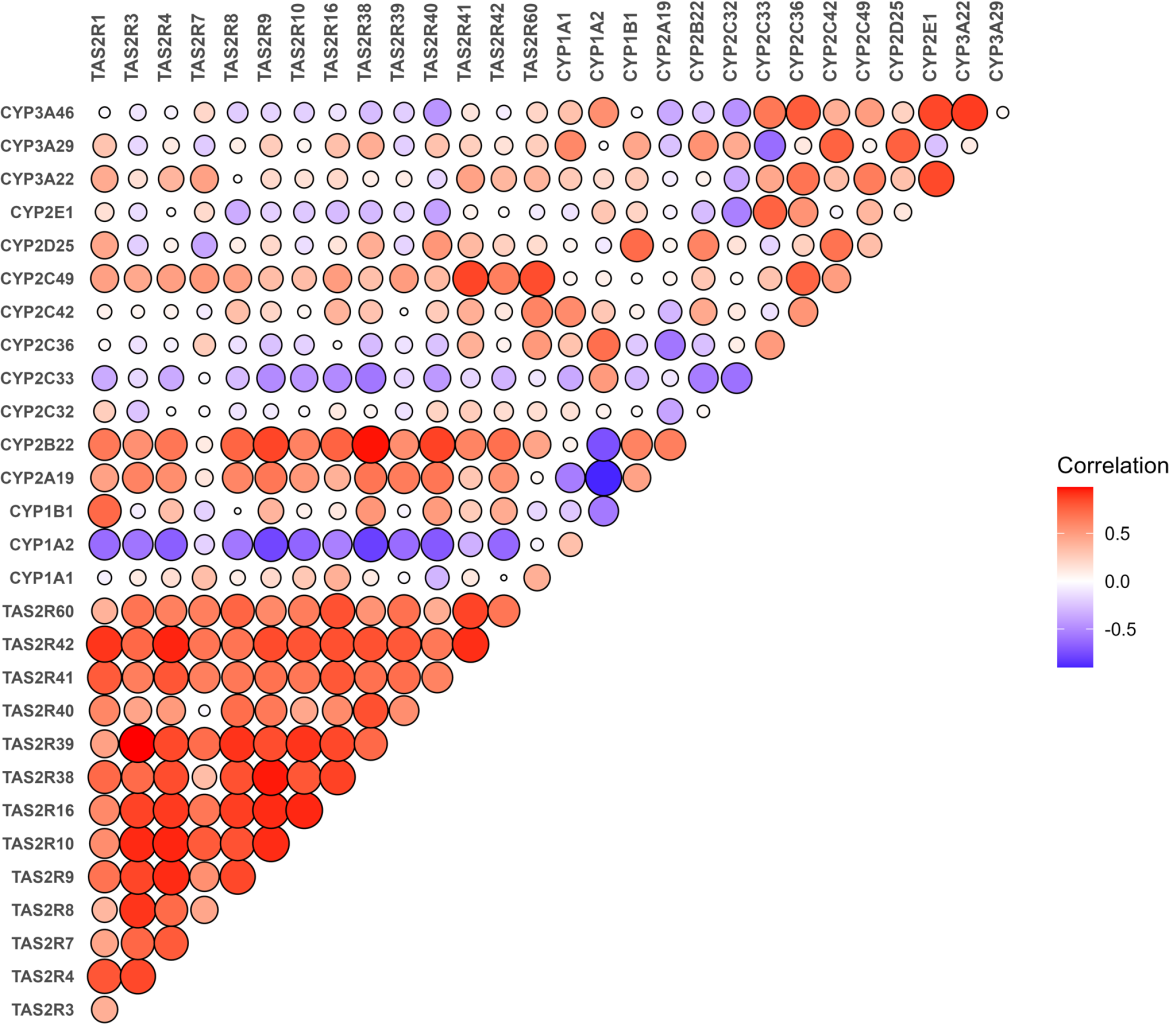
Correlation plot for cytochrome P450 enzymes and bitter taste receptors in liver. Data obtained from transcriptomics (*n* = 7)

To investigate potential interactions between bitter taste receptors and cytochrome P450 enzymes involved in detoxification, we analysed correlations between TAS2R gene expression and CYP family members 1–3. Data were obtained from transcriptomic analysis from seven liver samples. Our correlation analysis revealed distinct patterns of co-expression between specific TAS2Rs and CYP isoforms (Fig. 7). For example, CYP1A2 exhibited a strong negative correlation with all bitter taste receptors except TAS2R7 and TAS2R60. CYP2A19 and CYP2B22 showed strong positive correlations with all TAS2Rs, except for TAS2R7 and, in the case of CYP2A19, also TAS2R60. Meanwhile, CYP2C49 and CYP2C42 displayed moderate positive correlations with all TAS2Rs, whereas CYP2C33 showed moderate negative correlations with all TAS2Rs.

## Discussion

Bitter taste receptors, known as TAS2Rs, have emerged as intriguing targets within the pharmaceutical market due to their involvement in various metabolic and physiological processes beyond their traditional role in the oral cavity (Lu et al. 2017; Ekstrand et al. 2017). Recent advances in taste receptor biology further highlight the potential of using TAS2Rs as therapeutic targets or predictive markers for treatment responses in various disorders. These receptors are believed to contribute to the side effects of many drugs, particularly as a substantial 30% of drugs target GPCRs, a large number of which are associated with bitter taste (Clark et al. 2012). While most studies on bitter taste receptors have been conducted in rodents and humans, research using the pig model remains significantly underexplored. This work aims to bridge that gap by investigating the expression of TAS2Rs in various porcine tissues.

The present study demonstrates that bitter taste receptors are expressed not only in oral tissues but also in various extra-oral porcine tissues. Specifically, in pigs, we identified the presence of 8 TAS2Rs across a range of tissues, including the tongue, liver, kidney, white adipose tissue, jejunum, duodenum, ileum, and colon. While previous studies focused on the expression in porcine circumvallate papillae (Da Silva et al. 2014) our research provides novel information about the tissues that have not been examined before. We identified that expression level of receptor may differ from tissue to tissue. Specifically, our data show the significant difference in expression level between white adipose tissue, liver and kidney. Adipose tissue is a metabolically active organ primarily responsible for energy storage, but it also functions as an endocrine organ, producing biologically active compounds that regulate metabolic balance. It is increasingly recognized as a key component of a complex network that influences various biological processes, including glucose and lipid metabolism, body weight regulation, and appetite control (Coelho et al. 2013).

Our results showing the expression of multiple TAS2Rs in porcine white adipose tissue are consistent with findings by Kimura and Kato (2020), who reported the presence of eleven TAS2R members in mouse adipose tissue. Similarly, Cancello et al. (2020) demonstrated that TAS2R38 expression in both subcutaneous and visceral adipose tissue depots is increased in obese individuals compared to lean subjects. These parallels suggest a conserved role for TAS2Rs in adipose tissue function across species. Additionally, our analysis revealed no significant differences in the expression of TAS2Rs across the different regions of the small intestine (duodenum, jejunum, and ileum) or between the small intestine and the large intestine (colon). This suggests a relatively uniform distribution of TAS2R expression within the gastrointestinal system. Similarly, expression levels of TAS2Rs in the tongue and liver were comparable, with no significant variation between the two tissues, indicating that TAS2Rs are expressed at similar levels in both tissues under basic conditions. Among the analysed porcine tissues, TAS2R expression was lowest in the kidney. This pattern aligns with findings in mice, where only a limited subset of TAS2Rs, specifically TAS2R105 and TAS2R106, were detected in the kidney (Liu et al. 2015), suggesting that renal expression of TAS2Rs may be restricted or receptor-specific across species. Taken together, these findings suggest that the presence of bitter taste receptors outside the oral cavity is a widespread phenomenon across species.

To further investigate the relationships between TAS2Rs we performed Pearson's correlation analysis. The significant positive correlations between the TAS2R expression in tongue, liver, and white adipose tissue suggest that these three organs may be interconnected in their roles in nutrient sensing, metabolism, and energy regulation. The liver and white adipose tissue communicate through hepatokines and adipokines, which regulate fat storage, insulin sensitivity, and energy homeostasis, further linking their metabolic roles (Kato and Oshima 2023; Tattoli et al. 2024). The liver plays a crucial role in systemic metabolism and is closely linked to adipose tissues, regulating lipid storage and metabolism by controlling the flow of metabolites to white adipose tissue (WAT) and brown adipose tissue (BAT). Moreover, regulatory molecules produced in the liver have been shown to influence subclinical inflammation and insulin resistance in adipose tissue, both of which are significant characteristics of obesity and metabolic syndrome (Scheja and Heeren 2016). It has been suggested that expression of mTAS2R138 in mouse gut is regulated by SREBP-2 and upregulated by low-cholesterol diet, indicating a possible protective mechanism during plant-based diets—typically low in cholesterol and potentially rich in plant toxins (Jeon et al. 2008). It has been suggested that stimulation of bitter taste receptors could explain off-target effect of drugs which possess bitter taste and their potential role in new therapeutics targets (Clark et al. 2012). Studies have shown that the expression of hTAS2r38 gene could be associated with obesity development (Chupeerach et al. 2021), regulation of dietary intake as well as energy and adipose tissue metabolism (Choi 2019) or adipocytes differentiation and lipid breakdown (Cancello et al. 2020). Ortega et al. 2016 highlighted the influence of the human TAS2R38 gene on phenotypic and clinical outcomes related to obesity, showing that genes are significantly associated with extreme weight conditions, such as obesity and anorexia nervosa. These findings emphasize the potential of targeting adipose-liver communication to develop new therapeutic strategies for metabolic disorders such as obesity, type 2 diabetes, and metabolic dysfunction-associated fatty liver disease (MAFLD). To our knowledge, no prior research has documented these specific receptor correlations across these tissues, making this observation a significant contribution to the understanding of TAS2Rs beyond their traditional role in taste perception. Interestingly, we observed significant positive correlations for TAS2R1 expressed in the duodenum and tongue. The duodenum, the first and shortest segment of the small intestine, plays a crucial role in digestion by preparing chyme for nutrient absorption. It accomplishes this by introducing bile from the gallbladder and digestive enzymes from the pancreas. Bile acids, synthesized in the liver and released into the proximal duodenum, are integral to lipid digestion and absorption, breaking down fats into smaller molecules that can be efficiently absorbed further along the gastrointestinal tract (Farre et al. 2020). Recent studies have highlighted the potential role of TAS2Rs in sensing bile acids, extending the known functions of these receptors beyond taste perception (Ekstrand et al. 2017). A study by Ziegler et al., (2023) demonstrated that several human TAS2Rs, including TAS2R1, TAS2R4, TAS2R14, TAS2R39, and TAS2R46 expressed in small intestine are responsive to at least three bile acids.

Conversely, negative correlations were evident between the expression of TAS2Rs in ileum and colon. The ileum, as the final segment of the small intestine, plays a crucial role in absorbing nutrients, particularly vitamin B12 and bile salts, which are necessary for the digestion of fats (Grüner and Mattner 2021). The colon plays a crucial role in digestion, absorbing water and electrolytes from undigested food to form solid feces. Additionally, the colon reabsorbs bile acids that aid fat digestion, with some being modified by gut bacteria into secondary bile acids. These bile acids contribute to fat absorption, metabolic regulation, and influence gut microbiome composition (Cai et al. 2022). The negative correlation between the bitter taste receptors expression in ileum and colon could be related to differences in their functions, bile acid regulation, and microbiota interactions. Both parts of the intestines are involved in bile acid metabolism, but their roles diverge significantly. The ileum is primarily responsible for the reabsorption of bile acids, which are then returned to the liver through the enterohepatic circulation, while the colon plays a role in modifying these bile acids into secondary bile acids through gut microbiota activity. This suggests that the negative correlation between the two could be influenced by distinct biochemical environments in each region, with TAS2Rs potentially playing different roles in metabolic regulation and bile acid processing. The negative correlation between the ileum and white adipose tissue may stem from the distinct physiological and metabolic roles these two regions play in the body. The significant differences in expression observed in the ddPCR analysis between WAT, tongue and liver are further supported by the positive correlations found in the Pearson’s correlation analysis, indicating a possible link between receptor expression in these tissues.

Regarding bitter taste receptors, analysis revealed the presence of 16 TAS2R transcripts in both, tongue and liver tissues. However, no statistically significant alterations were observed. Although transcriptomic analysis did not indicate significant changes, it confirmed TAS2Rs presence at the transcript level. This finding aligns with previous reports from Lai et al. (2023) who identified thirteen transcripts encoding bitter taste receptors in the circumvallate papillae of pigs. To date, there has been no further research comparing the differences in TAS2R expression between the tongue and liver, nor exploring the potential implications of these differences.

## Conclusions

Our study, for the first time, identified the expression of eight TAS2Rs at the mRNA level in porcine oral and extra-oral tissues. We investigated several tissues (liver, kidney, white adipose tissue, jejunum, duodenum, colon, and ileum) that have not been previously examined for TAS2R expression. Notably, strong positive correlations in the expression of TAS2R1, TAS2R4, TAS2R10, TAS2R38, TAS2R40, and TAS2R42 across the tongue, liver, and adipose tissue suggests a possible interconnection between these organs in metabolism, energy regulation, and bile acid function. Moreover, the strong correlation observed between members of the cytochrome P450 family (CYP1A2, CYP2C33, CYP2B22, CYP2A19, CYP2C49, and CYP2C42) and all bitter taste receptors in the liver may indicate a link between TAS2Rs and the detoxification and metabolism of xenobiotics.

Our findings underscore the value of examining TAS2R expression patterns across metabolically active tissues. Although transcriptomic analysis did not reveal statistically significant differences under the tested conditions, the confirmed presence of multiple TAS2Rs at the transcript level supports their relevance beyond taste perception.

## Supplementary Information

Below is the link to the electronic supplementary material.

Supplementary file 1 (DOCX 184 KB)

## Data Availability

All data and materials generated in this study are available upon request.
