# Supplementary material for: Extra-oral expression of bitter taste receptors in pigs and their correlation with hepatic cytochrome P450 enzymes
Source: Cell Tissue Res. 2025 Nov 5;402(3):255–65. doi: 10.1007/s00441-025-04021-w (PMC12727863; doi:10.1007/s00441-025-04021-w)
Supplement: Supplementary file 1 — (DOCX 184 KB) [file 441_2025_4021_MOESM1_ESM.docx]

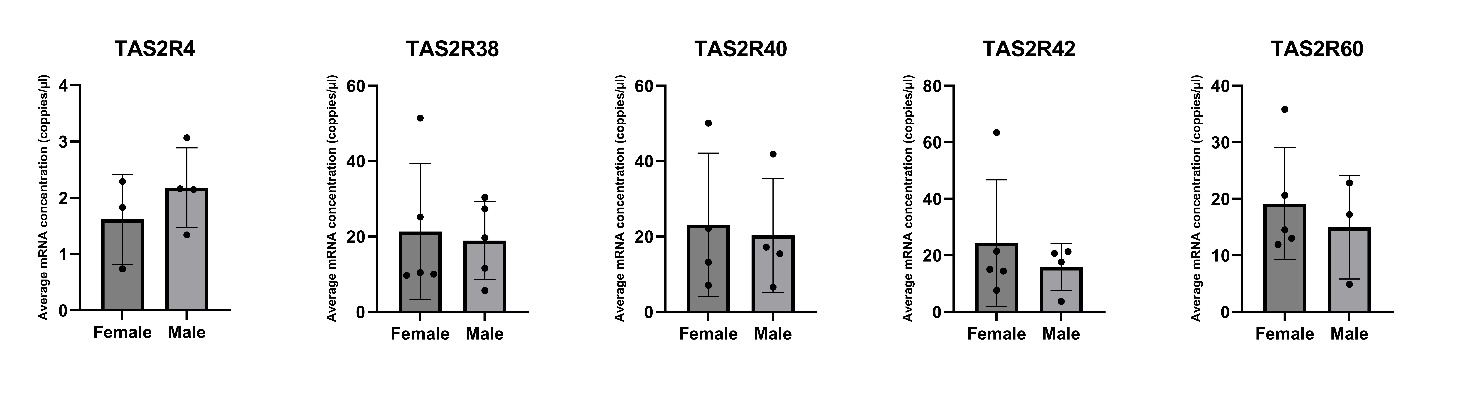


**Supplementary Figure 1**. Average mRNA concentration (coppies/µl) of bitter taste receptors in Göttingen minipigs’ liver. (n=3-5). Only mRNA amplified in at least three donors is shown in the graph. Data are presented as mean ± standard deviation. Statistical differences between sexes were assessed using an unpaired Students t-test.


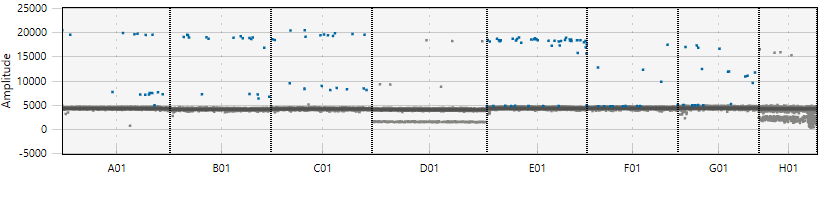


TAS2R1

58 57,5 56,5 55 53,2 51,7 50,6 50


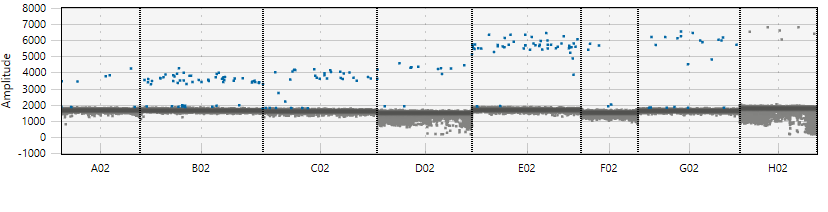


TAS2R4

58 57,5 56,5 55 53,2 51,7 50,6 50


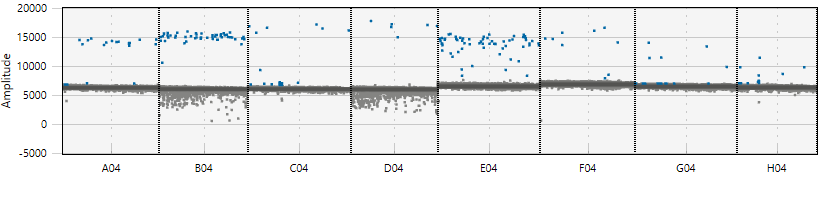


TAS2R38

TAS2R10

58 57,5 56,5 55 53,2 51,7 50,6 50


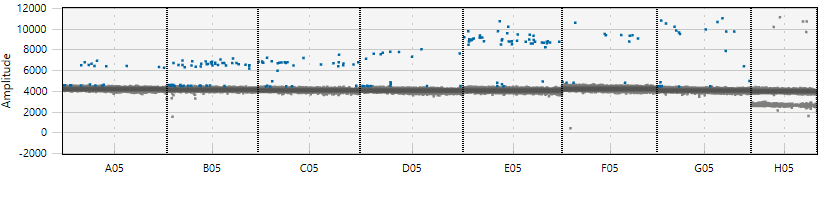


TAS2R40

58 57,5 56,5 55 53,2 51,7 50,6 50


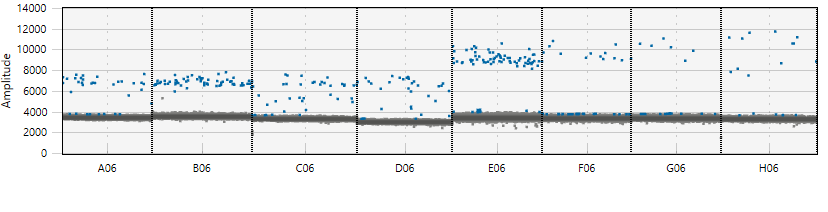


TAS2R42

58 57,5 56,5 55 53,2 51,7 50,6 50


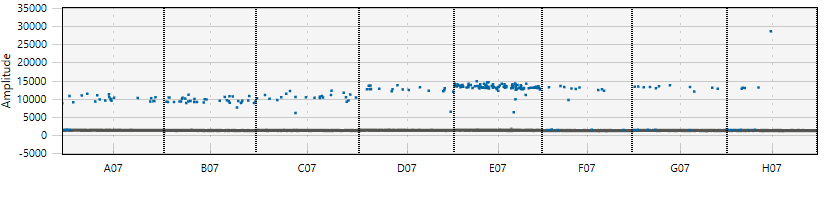


58 57,5 56,5 55 53,2 51,7 50,6 50

**Supplementary Figure 2.** Temperature gradient results to assess the optimal temperature in which the primer/probe had the best amplitude response during the annealing step of the thermal cycling process, targeting TAS2R1, TAS2R4, TAS2R10, TAS2R38, TAS2R40 and TAS2R42 genes. Consequently, the annealing temperature of the incoming experiments was set at 53 ºC.
